# Supplementary material for: Targeted profiling of human extrachromosomal DNA by CRISPR-CATCH
Source: Nat Genet. 2022 Oct 17;54(11):1746–54. doi: 10.1038/s41588-022-01190-0 (PMC9649439; doi:10.1038/s41588-022-01190-0)
Supplement: Supplementary file 1 — Supplementary Methods, Table 1 and References. [file 41588_2022_1190_MOESM1_ESM.pdf]

nature genetics

Technical Report

https://doi.org/10.1038/s41588-022-01190-0

Targeted profiling of human extrachromosomal DNA by CRISPR-CATCH

In the format provided by the authors and unedited

SUPPLEMENTARY INFORMATION

Targeted profiling of human extrachromosomal DNA by CRISPR-CATCH

King L. Hung, Jens Luebeck, Siavash R. Dehkordi, Caterina I. Colón, Rui Li, Ivy Tsz-Lo Wong, Ceyda Coruh, Prashanthi Dharanipragada, Shirley H. Lomeli, Natasha E. Weiser, Gatien Moriceau, Xiao Zhang, Chris Bailey, Kathleen E. Houlahan, Wenting Yang, Rocío Chamorro González, Charles Swanton, Christina Curtis, Mariam Jamal-Hanjani, Anton G. Henssen, Julie A. Law, William J. Greenleaf, Roger S. Lo, Paul S. Mischel, Vineet Bafna, Howard Y. Chang*

* Correspondence should be addressed to: howchang@stanford.edu

Table of Contents

- Supplementary Methods
- Supplementary Table 1
- Supplementary References

SUPPLEMENTARY METHODS

Amplicon reconstruction from CRISPR-CATCH sequencing

Using short-read sequencing data from CRISPR-CATCH with double size selection as described above, we implemented new strategies and modified existing methods[1] to resolve ecDNA structures. Broadly, the methods involved seven steps. The last six steps are available in a CRISPR-CATCH reconstruction pipeline, available at https://github.com/siavashre/CRISPRCATCH.

1. To identify the regions of interest, we ran PrepareAA (https://github.com/jluebeck/PrepareAA) (version 0.931.4) and AmpliconArchitect (version 1.2_r2, available from https://github.com/jluebeck/AmpliconArchitect) on two public bulk SNU16 WGS datasets (SRX5466612[2]; SRR530826, Genome Research Foundation) and found comparable graphs in both. We used PrepareAA with BWA-MEM[3] (version 0.7.12-r1039) to align reads to hg19 and CNVKit[4] (version 0.9.7) to generate seed regions having copy number (CN) > 5. These regions were provided to AA, which constructed a CN-aware breakpoint graph. The genome regions AA included in the graph were converted to bed format and used as the seed regions in the analysis of each PFGE band, so that the regions studied were always consistent between bands.

2. Using WGS reads generated from CRISPR-CATCH-isolated DNA, for each band we next aligned to the hg19 reference genome using PrepareAA which included BWA MEM and a PCR-duplicate removal step (using samtools[5] version 1.3.1), and we also made estimates of insert size distribution using Picard (version 2.25.6) for quality control purposes.

3. The aligned PFGE data and seed regions identified from bulk sequencing were provided to AmpliconArchitect (version 1.2_r2) to construct the CN-aware breakpoint graph, using non-default arguments –downsample -1 –pair_support 2 –no_cstats –insert_sdevs 8.5. The –insert_sdevs parameter allows for larger insert size variation without forming breakpoints from read pairs marked as discordant, as we found high insert size variance occurred frequently in DNA extracted from the gels. Following AA, we

ran a script on the resulting CN-aware breakpoint graph to filter non-foldback graph edges joining regions smaller than 1 kb from the graph, representing potential unfiltered artifact edges arising from overdispersion in insert size variance, in order to reduce the complexity of the graph when performing pathfinding. Since the edges removed joined regions not more than 1 kb apart and did not lead to changes in the orientation of the genome, this step had a negligible effect on the resulting paths. This utility for filtering AA graphs is made available as part of PrepareAA (graph_cleaner.py).

4. Central to the method for ecDNA reconstruction is the assumption that a single ecDNA is being analyzed within the graph, and as a result the estimated genomic copy numbers should closely relate to the number of times a segment appears within the ecDNA. We termed the number of times a segment appeared within a single ecDNA as the “multiplicity” of a genomic segment. The path finding method first removes low CN elements from the graph representing the background genome and contamination from incomplete separation of ecDNAs (i.e., remove segments with CN below 20% of the maximum CN of all segments having length > 100 bp, or below 10% of the maximum, if the maximum CN is >10000). In the remaining segments, we assumed that the majority of segments appeared once within an ecDNA. We assumed that ecDNAs for which the majority of segments are present more than once would reflect cases where two or more ecDNAs were present, instead of one. Thus, to compute the multiplicity of each graph segment, the method computes the 40th percentile of the remaining graph segment copy numbers and assigns that copy number, S_1, to multiplicity = 1. For each segment, *i*, in the graph, we computed its multiplicity, *M(i)* as.

$$M(i) = \mathrm{round}(\frac{CN(i)}{S_1})$$

5. To find paths in the graph which represented candidate ecDNA structures, we used an exhaustive search constrained by the multiplicities of the segments and (if available) the estimated maximum molecule size suggested by the CRISPR-CATCH data. Candidate ecDNA structures are determined through a constrained depth-first search (DFS) approach, which attempts to identify paths in the graph, and performs the process starting

at every segment in the graph assigned a non-zero multiplicity. During the search, the length of the path (in base pairs) must remain less than the maximum allowed length (L). For every segment i, appearing n_i times in the path, $n_i \leq M(i)$. The DFS recursion terminates if either constraint is violated, and the current path is scored as $\sum_i n_i$. The path is compared against the current best path (initiated as an empty path with score 0) and updated if it scores higher. Both the best-scoring cyclic paths as well as the best-scoring paths regardless of cyclic status are returned after removing all duplicate (identical) paths from the collection of best-scoring paths. This utility is also individually available from PrepareAA (plausible_paths.py).

6. We found a number of features of both the breakpoint graph and the reconstructions to be informative about the quality of the data in the band. We developed quality annotations reported along with each reconstruction to provide users with annotations about the confidence of the reconstruction. We note that CN-aware breakpoint graphs derived from NGS data may contain a number of error sources including missing edges between graph segments and incorrect estimation of copy numbers (leading sometimes to incorrect estimation of multiplicity). The method applies the following filters.

a) In the amplicon region analyzed by AA, the total amount of amplified material (non-zero multiplicity) should not significantly exceed the maximum estimated molecular size of the band (if provided). We used a cutoff such that amplicons with 1.4x the maximum estimated molecular size of the band were flagged for low quality (incomplete separation of ecDNA).

b) Changes in multiplicity must be accompanied by one or more breakpoint junctions, and thus for a breakpoint graph with $|e|$ total edges, amplicons where

$$\frac{|e|}{\max(M(i))} < 1$$

were flagged for low quality (missing graph edges).

c) We defined a root mean square residual for the unexplained copy numbers of *M(i)*. In a given path, for each segment *i*, having n_i occurrences in the path, the root mean square residual was defined as

$$RMSR = \sqrt{\frac{1}{N}\sum_{i=1}^{N}\left(n_i - M(i)\right)^2}$$

where *N* is the number of segments having non-zero multiplicity in the graph. We set a default cutoff such that amplicons with RMSR > 0.9 were flagged as low quality (too many amplified graph segments having incompletely used multiplicity).

d) To assess how tightly segment copy numbers could be segregated by segment multiplicity, we computed the Davies-Bouldin index[6] (DBI) on the clusters of copy numbers. Each cluster was comprised of all segment copy numbers assigned to a multiplicity (singleton clusters excluded), and the centroid of the cluster was the mean CN for the cluster. Amplicons where the DBI was > 0.3 were flagged as low quality due to noisy copy number estimation.

e) If a minimum molecular size for the band was given, we flagged reconstructions which fell below that 90% of that value as low quality as they reflected incomplete reconstructions.

f) If no segment in the reconstruction overlapped the CRISPR-Cas9 target site, we flagged it as being low quality as it was either an incomplete reconstruction, or the incorrect amplicon was detected.

7. Since the reconstructed paths are reported in the textual AA_cycles.txt format, the method also provides automated circular visualizations of the structures and the WGS coverage tracks which are generated by CycleViz (https://github.com/jluebeck/CycleViz) (version 0.1.0).

Optical mapping

Optical maps from SNU16 cells were generated as follows: ultra-high molecular weight (UHMW) DNA was extracted from 1.5 million frozen cells preserved in DMSO following the manufacturer's instructions (Bionano Genomics, #30398, 80042). Briefly, cells were digested with Proteinase K (Puregene #158920) and RNAse A (Puregene #158922) and then the DNA was precipitated with isopropanol and bound with nanobind magnetic disks. Bound UHMW DNA was resuspended in elution buffer (EB) and quantified with Qubit dsDNA BR assay kit (ThermoFisher Scientific, Q32850). The final DNA concentration was initially too high (280 ng/μl), therefore UHMW DNA was further diluted with EB, gently mixed with a wide-bore tip five times, and allowed to relax at room temperature for two days. Upon resting, UHMW DNA was diluted to 110 ng/μl in EB and DNA labeling was performed following the manufacturer's instructions (Bionano Genomics, #30206, 80005). Standard Direct Labeling Enzyme 1 (DLE-1) reactions were carried out using 750 ng of purified UHMW DNA. Using the Qubit dsDNA HS assay kit (ThermoFisher Scientific, #32854), the final labeled DNA concentration was determined as 5.4 ng/μl with a coefficient of variation of 0.026. The fluorescently labeled DNA molecules were loaded onto the Saphyr Chip G2.3 (Bionano Genomics, #20366, 30142) and were imaged sequentially across nanochannels on a Saphyr instrument (Bionano Genomics, #90023). An effective genome coverage of approximately 340X, using molecules >= 150 kb (molecule N50 of 0.2505 Mb) was achieved.

De novo assembly of SNU16 was performed with Bionano's *de novo* assembly pipeline (Bionano Solve v3.6, #90023) using standard haplotype aware arguments. With the Overlap-Layout-Consensus paradigm, pairwise comparison of DNA molecules of approximately 130X coverage was used to create a layout overlap graph, which was then used to generate the initial consensus genome maps, which had a contig N50 of 50 Mb. By realigning molecules to the genome maps (P value cutoff of $<10^{-12}$), and by using only the best matched molecules, a refinement step was done to refine the label positions on the genome maps and to remove chimeric joins. Next, during an extension step, the software aligned molecules to genome maps ($P<10^{-12}$), and extended the maps based on the molecules aligning past the map ends. Overlapping genome maps were then merged

($P<10^{-16}$). These extension and merge steps were repeated five times before a final refinement ($P<10^{-12}$) was applied to “finish” all genome maps.

Validating candidate structures with optical mapping

To validate candidate ecDNA paths we used long-range optical mapping (OM) data. Previously, we developed a method, AmpliconReconstructor (AR)[7], which uses OM data and AA’s outputs as inputs. AR attempts to identify paths within the breakpoint graph supported by OM contigs. In the CRISPR-CATCH data, the graphs may have many smaller segments than the graphs derived from bulk WGS, due to noisier CN profiles in the gel-extracted DNA, or due to highly complex ecDNA structures having very dense breakpoints. In these cases, large numbers of graph segments may be too small to reliably align against OM contigs using AR’s standard approach. Thus, we added a method to AR whereby the user can provide an AA-formatted cycles.txt file containing the full candidate path. The individual segments are combined into a single long genome sequence, then converted to an *in silico* digested OM sequence. The combined candidate OM sequence is then aligned with OM contigs using AR’s SegAligner method. The resulting candidate sequence alignment label indices are mapped back to the original uncombined candidate path. Contig alignments for the candidate path were combined if necessary and visualized using CycleViz. The candidate path alignment utility added to AR for this analysis is available at https://github.com/jluebeck/AmpliconReconstructor.

ChIP-seq

ChIP-seq data for SNU16 were previously published under GEO accession GSE159986[8]. Paired-end reads were aligned to the hg19 genome using Bowtie2[9] (version 2.3.4.1) with the --very-sensitive option following adapter trimming with Trimmomatic[10] (version 0.39). Reads with MAPQ values less than 10 were filtered using samtools (version 1.9) and PCR duplicates removed using Picard’s MarkDuplicates (version 2.20.3-SNAPSHOT). ChIP-seq signal was converted to bigwig format for visualization using deepTools bamCoverage[11] (version 3.3.1) with the following parameters: --bs 5 --smoothLength 105 --normalizeUsing CPM --scaleFactor 10.

Identification of SNU16 connected ecDNA segments

To identify enriched ecDNA species in SNU16 from CRISPR-CATCH, we obtained raw sequencing counts in all CRISPR-CATCH-isolated ecDNA species in 5-kb genomic bins using bedtools (version 2.30.0)[12], divided each count by total reads and multiplied by one million to give a normalized count (count per million, CPM). We then calculated the log2 fold change of each bin in each sequencing library over WGS by dividing the respective CPMs followed by log-transformation. To map connected ecDNA segments in SNU16 using CRISPR-CATCH, we assigned a value of 1 to the 5-kb genomic bins with log2 fold changes over WGS above 3.5 and a value of 0 otherwise. For each pair of 5-kb genomic bins, co-occurrence was calculated by number of co-occurring 1's in each sequencing library across all CRISPR-CATCH-isolated SNU16 ecDNAs. To compare connected ecDNA segments identified by CRISPR-CATCH with chromatin conformation capture background signals, H3K27ac HiChIP count matrix for SNU16 was obtained from a previously published study under GEO accession GSE159986[8]. The Juicer Tools[13] (1.9.9) dump command was used to extract the chromosome of interest from the .hic file with 5-kb resolution without normalization and the unnormalized interaction counts were plotted in R. To predict connected ecDNA segments in bulk WGS, we ran PrepareAA (version 0.931.4) and AmpliconArchitect (version 1.2_r2) as described above on SNU16 WGS obtained from SRX5466612[2] to generate an amplicon cycles text file. Connected DNA segments were divided into 5-kb bins as before and co-occurrences of bins on amplicons were summed and plotted in R to compare with the CRISPR-CATCH result and chromatin conformation capture interaction signals.

Supplementary Table 1.

gRNA sequence	gRNA information
TGGCGCAGTTATGCTTTAAC	*EGFR* guide A, used in GBM39 experiments
GGATCTACTTGGCACTCGCT	*EGFR* guide B, used in GBM39 experiments
CAATACCGCACTCAATGTCA	*EGFR* guide C, used in GBM39 experiments
ACAAACCGCGAGATCAGGGG	*EGFR* guide D, used in GBM39 experiments
ACGTTAAAAAGCTGTCGCGC	*EGFR* guide E, used in GBM39 experiments
TCCCGTGCGCGATGACGACA	*EGFR* guide F, used in GBM39 experiments
TAAACCACGGAAGCGGCGGC	*EGFR* guide G, used in GBM39 experiments
GCCTTGTCGTCATCGCGCAC	*EGFR* guide H, used in GBM39 experiments
CCAGCAATCGTTAACCACTG	*MYC* guide 3, used in SNU16 experiments
CTTCGGGGAGACAACGACGG	*MYC* guide 5, used in SNU16 experiments
GTGATATTTGAACCGCCCTG	*MYC* guide 7, used in SNU16 experiments
GGGGATTGGTACCGTAACCA	*FGFR2* guide 17, used in SNU16 experiments
GAGGCGATAATATCAACATG	*FGFR2* guide 18, used in SNU16 experiments
ATCATGTAGTATCCCCCACC	*MYC* guide 82, used in SNU16 experiments
AAGCGGTTTAAATACAGCGC	SNU16 guide targeting enhancer E1
CCTAGGTTTTACGCATTCAT	SNU16 guide targeting enhancer E2
TTAAGCGCGCGGCGGCAGCA	SNU16 guide targeting enhancer E3
GGGTGTTAACCGTAGGATGA	SNU16 guide targeting enhancer E4
ACGAAGCCCATACATAAGGT	SNU16 guide targeting enhancer E5
CCAGTGTGCACCTTACCCGG	SNU16 guide targeting enhancer E6
CGTAGCTACATGTCTCATAG	*NRAS* guide 194, used in patient tumor experiment

SUPPLEMENTARY REFERENCES

1. Deshpande, V. *et al.* Exploring the landscape of focal amplifications in cancer using AmpliconArchitect. *Nature Communications* **10**, 392 (2019).
2. Ghandi, M. *et al.* Next-generation characterization of the Cancer Cell Line Encyclopedia. *Nature* **569**, 503–508 (2019).
3. Li, H. & Durbin, R. Fast and accurate short read alignment with Burrows–Wheeler transform. *Bioinformatics* **25**, 1754–1760 (2009).
4. Talevich, E., Shain, A. H., Botton, T. & Bastian, B. C. CNVkit: Genome-Wide Copy Number Detection and Visualization from Targeted DNA Sequencing. *PLOS Computational Biology* **12**, e1004873 (2016).
5. Li, H. *et al.* The Sequence Alignment/Map format and SAMtools. *Bioinformatics* **25**, 2078–2079 (2009).
6. Davies, D. L. & Bouldin, D. W. A cluster separation measure. *IEEE Trans Pattern Anal Mach Intell* **1**, 224–227 (1979).
7. Luebeck, J. *et al.* AmpliconReconstructor integrates NGS and optical mapping to resolve the complex structures of focal amplifications. *Nature Communications* **11**, 4374 (2020).
8. Hung, K. L. *et al.* ecDNA hubs drive cooperative intermolecular oncogene expression. *Nature* **600**, 731–736 (2021).
9. Langmead, B. & Salzberg, S. L. Fast gapped-read alignment with Bowtie 2. *Nature Methods* **9**, 357–359 (2012).
10. Bolger, A. M., Lohse, M. & Usadel, B. Trimmomatic: a flexible trimmer for Illumina sequence data. *Bioinformatics* **30**, 2114 (2014).
11. Ramírez, F. *et al.* deepTools2: a next generation web server for deep-sequencing data analysis. *Nucleic Acids Res* **44**, W160–W165 (2016).
12. Quinlan, A. R. & Hall, I. M. BEDTools: a flexible suite of utilities for comparing genomic features. *Bioinformatics* **26**, 841–842 (2010).
13. Durand, N. C. *et al.* Juicer Provides a One-Click System for Analyzing Loop-Resolution Hi-C Experiments. *Cell Systems* **3**, 95–98 (2016).
