# Supplementary material for: Targeted profiling of human extrachromosomal DNA by CRISPR-CATCH
Source: Nat Genet. 2022 Oct 17;54(11):1746–54. doi: 10.1038/s41588-022-01190-0 (PMC9649439; doi:10.1038/s41588-022-01190-0)
Supplement: Supplementary file 2 — Reporting Summary [file 41588_2022_1190_MOESM2_ESM.pdf]

nature portfolio

Corresponding author(s): Howard Y. Chang

Last updated by author(s): July 25, 2022

Reporting Summary

Nature Portfolio wishes to improve the reproducibility of the work that we publish. This form provides structure for consistency and transparency in reporting. For further information on Nature Portfolio policies, see our Editorial Policies and the Editorial Policy Checklist.

Statistics

For all statistical analyses, confirm that the following items are present in the figure legend, table legend, main text, or Methods section.

n/a	Confirmed	
☐	☒	The exact sample size (*n*) for each experimental group/condition, given as a discrete number and unit of measurement
☐	☒	A statement on whether measurements were taken from distinct samples or whether the same sample was measured repeatedly
☐	☒	The statistical test(s) used AND whether they are one- or two-sided<br>*Only common tests should be described solely by name; describe more complex techniques in the Methods section.*
☒	☐	A description of all covariates tested
☐	☒	A description of any assumptions or corrections, such as tests of normality and adjustment for multiple comparisons
☐	☒	A full description of the statistical parameters including central tendency (e.g. means) or other basic estimates (e.g. regression coefficient) AND variation (e.g. standard deviation) or associated estimates of uncertainty (e.g. confidence intervals)
☐	☒	For null hypothesis testing, the test statistic (e.g. *F*, *t*, *r*) with confidence intervals, effect sizes, degrees of freedom and *P* value noted<br>*Give P values as exact values whenever suitable.*
☒	☐	For Bayesian analysis, information on the choice of priors and Markov chain Monte Carlo settings
☒	☐	For hierarchical and complex designs, identification of the appropriate level for tests and full reporting of outcomes
☐	☒	Estimates of effect sizes (e.g. Cohen's *d*, Pearson's *r*), indicating how they were calculated

Our web collection on statistics for biologists contains articles on many of the points above.

Software and code

Policy information about availability of computer code

Data collection	Short-read sequencing of DNA isolated by CRISPR-CATCH<br>DNA libraries were sequenced on the Illumina Miseq, the Illumina Nextseq 550 or the Illumina NovaSeq 6000 platform.<br><br>Nanopore Sequencing and 5mC methylation calling<br>DNA isolated by CRISPR-CATCH was directly used without amplification for nanopore sequencing. Sequencing libraries were prepared using the Rapid Sequencing Kit (Oxford Nanopore Technologies, SQK-RAD004) according to the manufacturer's instructions. Sequencing was performed on a MinION (Oxford Nanopore Technologies).<br><br>Optical Mapping<br>The fluorescently labeled DNA molecules were loaded onto the Saphyr Chip G2.3 (Bionano Genomics, #20366, 30142) and were imaged sequentially across nanochannels on a Saphyr instrument (Bionano Genomics, #90023).<br><br>De novo assembly of SNU16 was performed with Bionano's de novo assembly pipeline (Bionano Solve v3.6, #90023) using standard haplotype aware arguments
Data analysis	Whole-Genome Sequencing<br>Reads were trimmed of adapter content with Trimmomatic24 (version 0.39), aligned to the hg19 genome using BWA MEM25 (0.7.17-r1188), and PCR duplicates removed using Picard's MarkDuplicates (version 2.25.3).<br><br>Analysis of TCGA ecDNA amplicon sizes<br>To obtain ecDNA intervals for TCGA tumors, we ran AmpliconClassifier (version 0.4.6; https://github.com/jluebeck/AmpliconClassifier) on AmpliconArchitect outputs published previously using WGS data2. ecDNA amplicon sizes were estimated by summing ecDNA amplicon interval sizes for each tumor.

Metaphase DNA FISH image analysis
Colocalization analysis for two-color metaphase FISH data for ecDNAs in SNU16 cells described in Extended Data Figure 9f was performed using Fiji (version 2.1.0/1.53c)41. Images were split into the two FISH colors + DAPI channels, and signal threshold set manually to remove background fluorescence. Overlapping FISH signals were segmented using watershed segmentation. Colocalization was quantified using the ImageJ-Colocalization Threshold program and individual and colocalized FISH signals were counted using particle analysis.

Genetic variant analyses
SVs from short-read sequencing were identified with DELLY44 (version 0.8.7; using Boost version 1.74.0 and HTSlib version 1.12) using the delly call command. BCF files were converted to VCF using bcftools view in Samtools45. VAFs were calculated using both imprecise and precise variants. Read alignment was visualized using Gviz in R.

SNVs were identified using GATK (version 4.2.0.0)46 from short-read sequencing data as follows. First, base quality score recalibration was performed on bam files (generated as described above) using gatk BaseRecalibrator followed by gatk ApplyBQSR. Covariates were analyzed using gatk AnalyzeCovariates. SNVs were called using gatk Mutect2 from the recalibrated bam files, and SNVs were filtered using gatk FilterMutectCalls. Finally, vcf files were converted to table format using gatk VariantsToTable with the following parameters: "-F CHROM -F POS -F REF -F ALT -F QUAL -F TYPE -GF AD -GF GQ -GF PL -GF GT". Mutation variant allele frequencies (VAFs) were calculated by dividing alternate allele occurrences by the sum of reference and alternate allele occurrences. SNVs which had coverage depth of 5 or less or were not detected in WGS were filtered out. Read alignment was visualized using Gviz in R. To classify ecDNA-specific SNVs in GBM39 cells, we identified all SNVs with VAFs higher than 0.03 in ecDNAs isolated by CRISPR-CATCH using guide A, B, or A+B (given chromosome contamination levels of 0.01-0.02; Extended Data Figure 2d) and with VAFs in WGS lower than 0.997 (non-homozygous variants). Chromosome-specific SNVs were defined as non-ecDNA SNVs with VAFs in WGS lower than 0.1. Homozygous SNVs were defined as non-ecDNA-specific and non-chromosome-specific SNVs with VAFs in WGS above 0.99.

Nanopore Sequencing and 5mC methylation calling
DNA isolated by CRISPR-CATCH was directly used without amplification for nanopore sequencing. Sequencing libraries were prepared using the Rapid Sequencing Kit (Oxford Nanopore Technologies, SQK-RAD004) according to the manufacturer's instructions. Sequencing was performed on a MinION (Oxford Nanopore Technologies).

Bases were called from fast5 files using guppy (Oxford Nanopore Technologies, version 5.0.16) within Megalodon (version 2.3.3) and DNA methylation status was determined using Rerio basecalling models with the configuration file "res_dna_r941_min_modbases-all-context_v001.cfg" and the following parameters: "--outputs basecalls mod_basecalls mappings mod_mappings mods per_read_mods --mod-motif Z CG 0 --write-mods-text --mod-output-formats bedmethyl wiggle --mod-map-emulate-bisulfite --mod-map-base-conv C T --mod-map-base-conv Z C". Methylation calls on single molecules were visualized using Integrative Genome Viewer (IGV, version 2.11.1) in bisulfite mode.

ATAC-seq
Adapter-trimmed reads were aligned to the hg19 genome using Bowtie2 (2.1.0). Aligned reads were filtered for quality using samtools (version 1.9)31, duplicate fragments were removed using Picard's MarkDuplicates (version 2.25.3), and peaks were called using MACS2 (version 2.2.7.1)32 with a q-value cut-off of 0.01 and with a no-shift model. Peaks from replicates were merged, read counts were obtained using bedtools (version 2.30.0)33 and normalized using DESeq2 (using the "counts" function in DESeq2 with normalized = TRUE; version 1.26.0)34.

MNase-seq
Reads were trimmed of adapter content with Trimmomatic24 (version 0.39), aligned to the hg19 genome using BWA MEM25 (0.7.17-r1188), and PCR duplicates removed using Picard's MarkDuplicates (version 2.25.3). Coverage of nucleosome midpoints was obtained using bamCoverage from deepTools (version 3.5.1) with the following parameters: "--MNase --binSize 1".

Amplicon reconstruction from CRISPR-CATCH sequencing
Using short-read sequencing data from CRISPR-CATCH with double size selection as described above, we implemented new strategies and modified existing methods6 to resolve ecDNA structures. Broadly, the methods involved seven steps. The last six steps are available in a CRISPR-CATCH reconstruction pipeline, available at https://github.com/siavashre/CRISPRCATCH.

1. To identify the regions of interest, we ran PrepareAA (https://github.com/jluebeck/PrepareAA) (version 0.931.4) and AmpliconArchitect (version 1.2_r2, available from https://github.com/jluebeck/AmpliconArchitect) on two public bulk SNU16 WGS datasets (SRX546661250; SRR530826, Genome Research Foundation) and found comparable graphs in both. We used PrepareAA with BWA-MEM37 (version 0.7.12-r1039) to align reads to hg19 and CNVKit51 (version 0.9.7) to generate seed regions having copy number (CN) > 5. These regions were provided to AA, which constructed a CN-aware breakpoint graph. The genome regions AA included in the graph were converted to bed format and used as the seed regions in the analysis of each PFGE band, so that the regions studied were always consistent between bands.

2. Using WGS reads generated from CRISPR-CATCH-isolated DNA, for each band we next aligned to the hg19 reference genome using PrepareAA which included BWA MEM and a PCR-duplicate removal step (using samtools45 version 1.3.1), and we also made estimates of insert size distribution using Picard (version 2.25.6) for quality control purposes.

3. The aligned PFGE data and seed regions identified from bulk sequencing were provided to AmpliconArchitect (version 1.2_r2) to construct the CN-aware breakpoint graph, using non-default arguments –downsample -1 –pair_support 2 –no_cstats –insert_sdevs 8.5. The –insert_sdevs parameter allows for larger insert size variation without forming breakpoints from read pairs marked as discordant, as we found high insert size variance occurred frequently in DNA extracted from the gels. Following AA, we ran a script on the resulting CN-aware breakpoint graph to filter non-foldback graph edges joining regions smaller than 1 kb from the graph, representing potential unfiltered artifact edges arising from overdispersion in insert size variance, in order to reduce the complexity of the graph when performing pathfinding. Since the edges removed joined regions not more than 1 kb apart and did not lead to changes in the orientation of the genome, this step had a negligible effect on the resulting paths. This utility for filtering AA graphs is made available as part of PrepareAA (graph_cleaner.py).

4. Central to the method for ecDNA reconstruction is the assumption that a single ecDNA is being analyzed within the graph, and as a result the estimated genomic copy numbers should closely relate to the number of times a segment appears within the ecDNA. We termed the number of times a segment appeared within a single ecDNA as the "multiplicity" of a genomic segment. The path finding method first removes low CN elements from the graph representing the background genome and contamination from incomplete separation of ecDNAs (i.e., remove segments with CN below 20% of the maximum CN of all segments having length > 100 bp, or below 10% of the maximum, if the

maximum CN is >10000). In the remaining segments, we assumed that the majority of segments appeared once within an ecDNA. We assumed that ecDNAs for which the majority of segments are present more than once would reflect cases where two or more ecDNAs were present, instead of one. Thus, to compute the multiplicity of each graph segment, the method computes the 40th percentile of the remaining graph segment copy numbers and assigns that copy number, S1, to multiplicity = 1. For each segment, i, in the graph, we computed its multiplicity, M(i) as.
$M(i)=\text{round}\left(\frac{CN(i)}{S_1}\right)$

5. To find paths in the graph which represented candidate ecDNA structures, we used an exhaustive search constrained by the multiplicities of the segments and (if available) the estimated maximum molecule size suggested by the CRISPR-CATCH data. Candidate ecDNA structures are determined through a constrained depth-first search (DFS) approach, which attempts to identify paths in the graph, and performs the process starting at every segment in the graph assigned a non-zero multiplicity. During the search, the length of the path (in base pairs) must remain less than the maximum allowed length (L). For every segment i, appearing ni times in the path, ni ≤ M(i). The DFS recursion terminates if either constraint is violated, and the current path is scored as $\sum_i n_i$. The path is compared against the current best path (initiated as an empty path with score 0) and updated if it scores higher. Both the best-scoring cyclic paths as well as the best-scoring paths regardless of cyclic status are returned after removing all duplicate (identical) paths from the collection of best-scoring paths. This utility is also individually available from PrepareAA (plausible_paths.py).

6. We found a number of features of both the breakpoint graph and the reconstructions to be informative about the quality of the data in the band. We developed quality annotations reported along with each reconstruction to provide users with annotations about the confidence of the reconstruction. We note that CN-aware breakpoint graphs derived from NGS data may contain a number of error sources including missing edges between graph segments and incorrect estimation of copy numbers (leading sometimes to incorrect estimation of multiplicity). The method applies the following filters.

a) In the amplicon region analyzed by AA, the total amount of amplified material (non-zero multiplicity) should not significantly exceed the maximum estimated molecular size of the band (if provided). We used a cutoff such that amplicons with 1.4x the maximum estimated molecular size of the band were flagged for low quality (incomplete separation of ecDNA).

b) Changes in multiplicity must be accompanied by one or more breakpoint junctions, and thus for a breakpoint graph with |e| total edges, amplicons where
$\frac{|e|}{\max(M(i))} < 1$
were flagged for low quality (missing graph edges).

c) We defined a root mean square residual for the unexplained copy numbers of M(i). In a given path, for each segment i, having n_i occurrences in the path, the root mean square residual was defined as
$RMSR=\sqrt{\frac{1}{N}\sum_{i=1}^{N}(n_i-M(i))^2}$
where N is the number of segments having non-zero multiplicity in the graph. We set a default cutoff such that amplicons with RMSR > 0.9 were flagged as low quality (too many amplified graph segments having incompletely used multiplicity).

d) To assess how tightly segment copy numbers could be segregated by segment multiplicity, we computed the Davies-Bouldin index52 (DBI) on the clusters of copy numbers. Each cluster was comprised of all segment copy numbers assigned to a multiplicity (singleton clusters excluded), and the centroid of the cluster was the mean CN for the cluster. Amplicons where the DBI was > 0.3 were flagged as low quality due to noisy copy number estimation.

e) If a minimum molecular size for the band was given, we flagged reconstructions which fell below that 90% of that value as low quality as they reflected incomplete reconstructions.

f) If no segment in the reconstruction overlapped the CRISPR-Cas9 target site, we flagged it as being low quality as it was either an incomplete reconstruction, or the incorrect amplicon was detected.

7. Since the reconstructed paths are reported in the textual AA_cycles.txt format, the method also provides automated circular visualizations of the structures and the WGS coverage tracks which are generated by CycleViz (https://github.com/jluebeck/CycleViz) (version 0.1.0).

Validating candidate structures with optical mapping
To validate candidate ecDNA paths we used long-range optical mapping (OM) data. Previously, we developed a method, AmpliconReconstructor (AR)7, which uses OM data and AA's outputs as inputs.

ChIP-seq
Paired-end reads were aligned to the hg19 genome using Bowtie253 (version 2.3.4.1) with the --very-sensitive option following adapter trimming with Trimmomatic36 (version 0.39). Reads with MAPQ values less than 10 were filtered using samtools (version 1.9) and PCR duplicates removed using Picard's MarkDuplicates (version 2.20.3-SNAPSHOT). ChIP-seq signal was converted to bigwig format for visualization using deepTools bamCoverage54 (version 3.3.1) with the following parameters: --bs 5 --smoothLength 105 --normalizeUsing CPM --scaleFactor 10.

Code availability
Custom code to perform reconstructions of candidate ecDNA structures from CRISPR-CATCH data is available at https://github.com/siavashre/CRISPRCATCH.

For manuscripts utilizing custom algorithms or software that are central to the research but not yet described in published literature, software must be made available to editors and reviewers. We strongly encourage code deposition in a community repository (e.g. GitHub). See the Nature Portfolio guidelines for submitting code & software for further information.

Data

Policy information about availability of data

All manuscripts must include a data availability statement. This statement should provide the following information, where applicable:

- Accession codes, unique identifiers, or web links for publicly available datasets
- A description of any restrictions on data availability
- For clinical datasets or third party data, please ensure that the statement adheres to our policy

Sequencing data generated in this study are deposited in SRA under BioProject accession PRJNA777710. WGS data from bulk GBM39 cells were obtained from the NCBI Sequence Read Archive, under BioProject accession PRJNA506071. WGS data from bulk SNU16 cells were previously generated (SRR530826, Genome Research Foundation). ATAC-seq and MNase-seq data for GBM39 were obtained from the NCBI Sequence Read Archive, under BioProject accession PRJNA506071. ChIP-seq data for SNU16 were previously published under GEO accession GSE15998628. Sequencing reads were mapped to the hg19 human reference genome. Source data are provided with this paper.

Field-specific reporting

Please select the one below that is the best fit for your research. If you are not sure, read the appropriate sections before making your selection.

☒ Life sciences ☐ Behavioural & social sciences ☐ Ecological, evolutionary & environmental sciences

For a reference copy of the document with all sections, see nature.com/documents/nr-reporting-summary-flat.pdf

Life sciences study design

All studies must disclose on these points even when the disclosure is negative.

Sample size	No sample size calculation was performed. Sample sizes for DNA sequencing, optical mapping and metaphase DNA FISH are consistent with current standard sample sizes in the published literature. Sample size for the patient sample was based on the available biological material.
Data exclusions	No data were excluded from analysis.
Replication	Method was performed using three or more independent biological replicates (including independent CRISPR guides and experiments) to capture variability. All replication attempts were successful.
Randomization	Randomization is not relevant to this study. Cell culture samples were collected without prior selection or bias and were randomly assigned to treatment or control conditions without prior selection or bias. Appropriate experimental controls are shown in figure panels.
Blinding	Blinding is not relevant to this study. All data were collected using instruments without bias. Furthermore, raw data for all experimental conditions were uniformly processed using the same data processing and analysis pipeline for each experiment, ensuring that no human bias is introduced in the data analysis.

Reporting for specific materials, systems and methods

We require information from authors about some types of materials, experimental systems and methods used in many studies. Here, indicate whether each material, system or method listed is relevant to your study. If you are not sure if a list item applies to your research, read the appropriate section before selecting a response.

Materials & experimental systems

n/a	Involved in the study	
☒	☐	Antibodies
☐	☒	Eukaryotic cell lines
☒	☐	Palaeontology and archaeology
☒	☐	Animals and other organisms
☒	☐	Human research participants
☒	☐	Clinical data
☒	☐	Dual use research of concern

Methods

n/a	Involved in the study	
☒	☐	ChIP-seq
☒	☐	Flow cytometry
☒	☐	MRI-based neuroimaging

Eukaryotic cell lines

Policy information about cell lines

Cell line source(s)	GBM39 neurospheres were derived from patient tissue as previously described (Wu et al. Nature. 2019). SNU16 cells were obtained from ATCC (CRL-5974).

Authentication	SNU16 cells were obtained from ATCC and therefore were not authenticated. GBM39 neurospheres were derived from patient tissue as previously described (Wu et al. Nature. 2019) and were authenticated using metaphase DNA FISH with probes hybridizing to EGFR as well as chromosome 7 centromeric probe to confirm ecDNA amplification status, same as in Wu et al. Nature. 2019.
Mycoplasma contamination	Cells were tested negative for mycoplasma.
Commonly misidentified lines (See ICLAC register)	None of the cell lines used are registered by ICLAC as commonly misidentified.
