# Supplementary material for: Targeted profiling of human extrachromosomal DNA by CRISPR-CATCH
Source: Nat Genet. 2022 Oct 17;54(11):1746–54. doi: 10.1038/s41588-022-01190-0 (PMC9649439; doi:10.1038/s41588-022-01190-0)

1. CHEF DNA Size Marker, 0.2–2.2 Mb, S. cerevisiae Ladder
2. CHEF DNA Size Marker, 1–3.1 Mb, H. wingei Ladder
3. no treatment
4. guide A
5. guide B
6. guide A+B
7. guide C+D
8. guide E+F
9. guide G+H

Raw image of PFGE agarose gel for CRISPR-CATCH for GBM39 cells. Image was cropped to remove extra white space and ladders, contrast was adjusted to make bands more visible. Corresponds to **Figure 1d**.

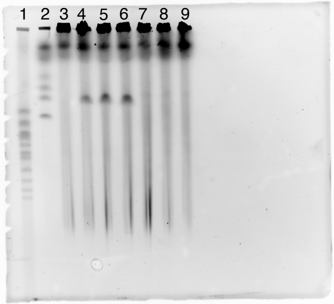

1. CHEF DNA Size Marker, 0.2–2.2 Mb, S. cerevisiae Ladder
2. CHEF DNA Size Marker, 1–3.1 Mb, H. wingei Ladder
3. GBM39 no treatment
4. GBM39 guide A
5. GBM39 guide E
6. GBM39 guide E+F
7. Jurkat no treatment
8. Jurkat guide A

Raw image of PFGE agarose gel for CRISPR-CATCH for indicated cell lines. Image was cropped to remove extra white space and ladders, contrast was adjusted to make bands more visible. Lanes 7/8 correspond to **Figure 1d**.

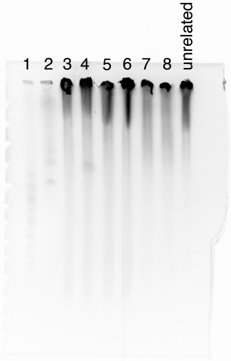

Supplement: Source Data Fig. 1 — Raw unprocessed PFGE images corresponding to Fig. 1d. [file 41588_2022_1190_MOESM8_ESM.pdf]
