# Supplementary material for: Targeted profiling of human extrachromosomal DNA by CRISPR-CATCH
Source: Nat Genet. 2022 Oct 17;54(11):1746–54. doi: 10.1038/s41588-022-01190-0 (PMC9649439; doi:10.1038/s41588-022-01190-0)
Supplement: Source Data Fig. 5 — Raw unprocessed PFGE images corresponding to Fig. 5b. [file 41588_2022_1190_MOESM10_ESM.pdf]

1. CHEF DNA Size Marker, 0.2–2.2 Mb, S. cerevisiae Ladder
2. CHEF DNA Size Marker, 1–3.1 Mb, H. wingei Ladder
3. no treatment
4. guide 7
5. guide 3
6. guide 5
7. guide 17
8. guide 18
9. guide 82

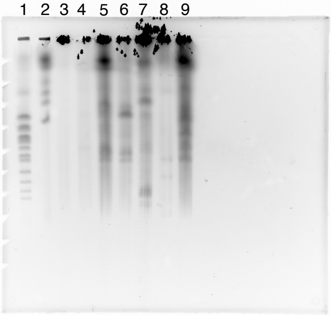

Raw image of PFGE agarose gel for CRISPR-CATCH for SNU16 cells. Image was cropped to remove extra white space and ladders, contrast was adjusted to make bands more visible. Corresponds to **Figure 5b**.
