# Supplementary material for: Targeted profiling of human extrachromosomal DNA by CRISPR-CATCH
Source: Nat Genet. 2022 Oct 17;54(11):1746–54. doi: 10.1038/s41588-022-01190-0 (PMC9649439; doi:10.1038/s41588-022-01190-0)

1. CHEF DNA Size Marker, 0.2–2.2 Mb, S. cerevisiae Ladder
2. CHEF DNA Size Marker, 1–3.1 Mb, H. wingei Ladder
3. GBM39 guide A CRISPR-CATCH
4. GBM39 no treatment CRISPR-CATCH
5. GBM39 in-solution HMW DNA isolation, no treatment

Raw image of PFGE agarose gel for CRISPR-CATCH and in-solution HMW DNA as indicated. Image was rotated and cropped to remove extra white space. Corresponds to **Extended Data Figure 1a**.

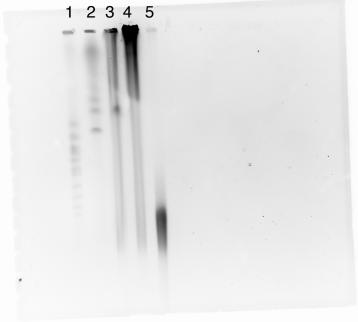

1. CHEF DNA Size Marker, 0.2–2.2 Mb, S. cerevisiae Ladder
2. CHEF DNA Size Marker, 1–3.1 Mb, H. wingei Ladder
3. GBM39 in-solution HMW DNA isolation, no treatment
4. GBM39 in-solution HMW DNA isolation, exonuclease

Raw image of PFGE agarose gel for in-solution HMW DNA as indicated. Image was cropped to remove extra white space and contrast was increased. Corresponds to **Extended Data Figure 1b**.

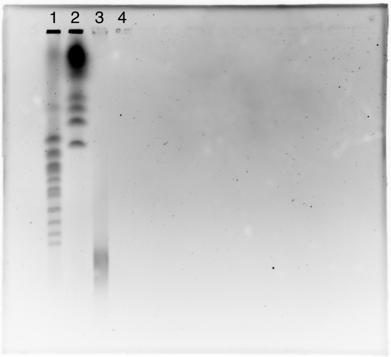

1. CHEF DNA Size Marker, 0.2–2.2 Mb, S. cerevisiae Ladder
2. CHEF DNA Size Marker, 1–3.1 Mb, H. wingei Ladder
3. no treatment

Raw image of PFGE agarose gel for untreated GBM39 cells. Image was cropped to remove extra white space. Corresponds to **Extended Data Figure 1d.**

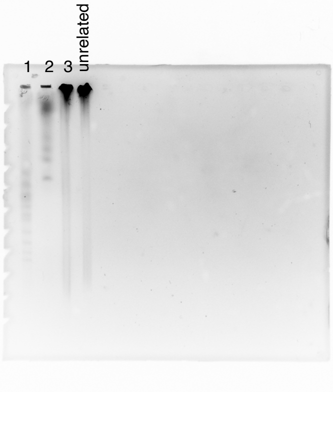

Supplement: Source Data Extended Data Fig. 1 — Raw unprocessed PFGE images corresponding to Extended Data Fig. 1a,b,d. [file 41588_2022_1190_MOESM11_ESM.pdf]
