# Supplementary material for: Targeted profiling of human extrachromosomal DNA by CRISPR-CATCH
Source: Nat Genet. 2022 Oct 17;54(11):1746–54. doi: 10.1038/s41588-022-01190-0 (PMC9649439; doi:10.1038/s41588-022-01190-0)

1. CHEF DNA Size Marker, 0.2–2.2 Mb, S. cerevisiae Ladder
2. CHEF DNA Size Marker, 0.2–2.2 Mb, S. cerevisiae Ladder + electrodepletion
3. CHEF DNA Size Marker, 1–3.1 Mb, H. wingei Ladder
4. CHEF DNA Size Marker, 1–3.1 Mb, H. wingei Ladder + electrodepletion

Raw image of PFGE agarose gel for size markers. Image was rotated and cropped to remove extra white space. Corresponds to **Extended Data Figure 3a.**

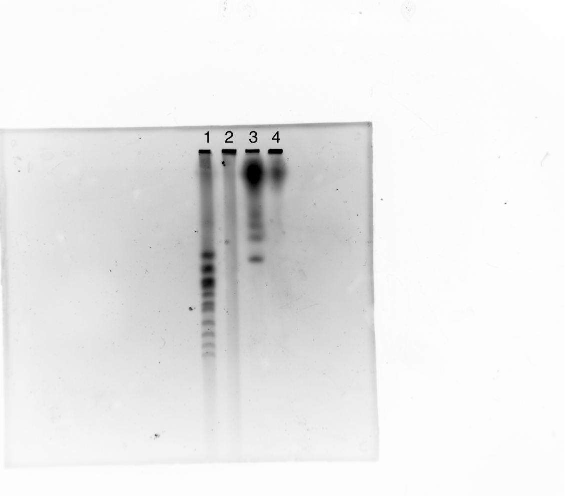

1. CHEF DNA Size Marker, 0.2–2.2 Mb, S. cerevisiae Ladder
2. CHEF DNA Size Marker, 1–3.1 Mb, H. wingei Ladder
3. SNU16 cells
4. SNU16 cells + electrodepletion
5. SNU16 cells + electrodepletion + guide 17

Raw image of PFGE agarose gel. Image was cropped to remove extra white space. Corresponds to **Extended Data Figure 3b.**

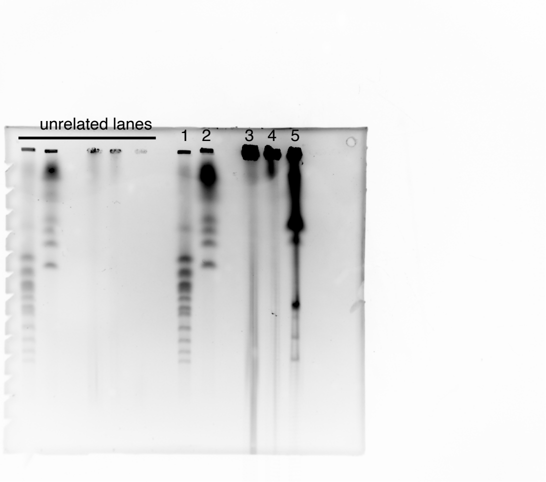

1. CHEF DNA Size Marker, 0.2–2.2 Mb, S. cerevisiae Ladder
2. CHEF DNA Size Marker, 1–3.1 Mb, H. wingei Ladder
3. melanoma patient Pt9 tumor + guide 194

Raw image of PFGE agarose gel for CRISPR-CATCH. Image was cropped to remove extra white space and ladders, contrast was increased. Corresponds to **Extended Data Figure 3e**.

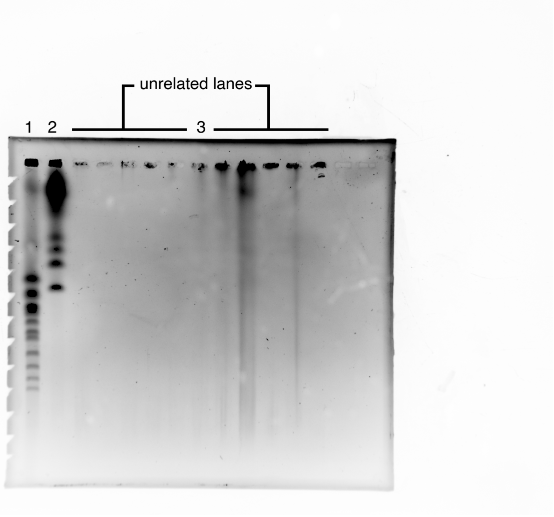

Supplement: Source Data Extended Data Fig. 3 — Raw unprocessed PFGE images corresponding to Extended Data Fig. 3a,b,e. [file 41588_2022_1190_MOESM12_ESM.pdf]
