# Supplementary material for: Targeted profiling of human extrachromosomal DNA by CRISPR-CATCH
Source: Nat Genet. 2022 Oct 17;54(11):1746–54. doi: 10.1038/s41588-022-01190-0 (PMC9649439; doi:10.1038/s41588-022-01190-0)
Supplement: Source Data Extended Data Fig. 8 — Raw unprocessed PFGE images corresponding to Extended Data Fig. 8c. [file 41588_2022_1190_MOESM13_ESM.pdf]

1. CHEF DNA Size Marker, 0.2–2.2 Mb, S. cerevisiae Ladder
2. CHEF DNA Size Marker, 1–3.1 Mb, H. wingei Ladder
3. no treatment
4. guide 17
5. guide 143
6. guide 146
7. guide 149
8. guide 152
9. guide 5
10. guide 155
11. guide 158

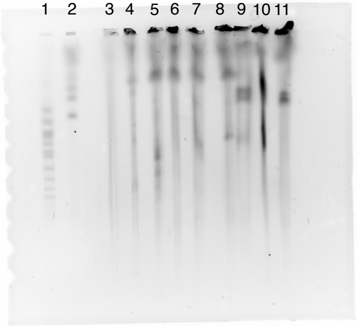

Raw image of PFGE agarose gel for CRISPR-CATCH for SNU16 cells. Image was rotated, cropped to remove extra white space and ladders, contrast was adjusted to make bands more visible. Corresponds to **Extended Data Figure 8c**.
